# Supplementary material for: Associations between sleep duration and insulin resistance in European children and adolescents considering the mediating role of abdominal obesity
Source: PLoS One. 2020 Jun 30;15(6):e0235049. doi: 10.1371/journal.pone.0235049 (PMC7326225; doi:10.1371/journal.pone.0235049)
Supplement: S1 Table — (DOCX) [file pone.0235049.s001.docx]

S1 Table: Model fit indices

|  | **Main model - whole group** | **Main model -  pre-school children** | **Main model - school children** | **Sensitivity analysis - additional adjustment for lifestyle factors** | **Sensitivity analysis - weekday nocturnal sleep duration** | **Sensitivity analysis - weekend nocturnal sleep duration** |
| --- | --- | --- | --- | --- | --- | --- |
|  | N=3 900 | N=863 | N=3 037 | N=3 239 | N=3 900 | N=3 900 |
| Chi-Square Test of Model Fit |  |  |  |  |  |  |
| Degrees of freedom | 3 | 3 | 3 | 3 | 3 | 3 |
| P-Value | 0.17 | 0.08 | 0.68 | 0.09 | 0.17 | 0.13 |
| Comparative Fit Index | 1.00 | 1.00 | 1.00 | 1.00 | 1.00 | 1.00 |
| Tucker-Lewis Index | 0.99 | 0.91 | 1.01 | 0.97 | 0.99 | 0.99 |
| Root Mean Square Error of Approximation | 0.013 | 0.038 | <0.001 | 0.019 | 0.013 | 0.015 |
|  | **Sensitivity analysis - complete case analysis - whole group** | **Sensitivity analysis - complete case analysis - pre-school children** | **Sensitivity analysis - complete case analysis - school children** | **Sensitivity analysis - HOMA-IR at baseline and/or follow-up - whole group** | **Sensitivity analysis - HOMA-IR at baseline and/ or follow-up - pre-school children** | **Sensitivity analysis - HOMA-IR at baseline and/or follow-up - school children** |
|  | N=1 319 | N=234 | N=1 085 | N=3 052 | N=594 | N=2 458 |
| Chi-Square Test of Model Fit |  |  |  |  |  |  |
| Degrees of freedom | 3 | 3 | 3 | 3 | 3 | 3 |
| P-Value | 0.27 | 0.21 | 0.36 | 0.14 | 0.07 | 0.29 |
| Comparative Fit Index | 1.00 | 1.00 | 1.00 | 1.00 | 1.00 | 1.00 |
| Tucker-Lewis Index | 0.99 | 0.88 | 1.00 | 0.99 | 0.86 | 1.00 |
| Root Mean Square Error of Approximation | 0.015 | 0.046 | 0.009 | 0.016 | 0.048 | 0.010 |

*HOMA-IR* homeostasis model assessment for insulin resistance
