# Supplementary material for: Associations between sleep duration and insulin resistance in European children and adolescents considering the mediating role of abdominal obesity
Source: PLoS One. 2020 Jun 30;15(6):e0235049. doi: 10.1371/journal.pone.0235049 (PMC7326225; doi:10.1371/journal.pone.0235049)
Supplement: S2 Table — (DOCX) [file pone.0235049.s002.docx]

**S2 Table:** Descriptive characteristics of the study population at baseline by abdominal obesity and insulin resistance status at baseline

|  | **Waist circumference <90^th^ percentile^a^**  **N=2 868** | **Waist circumference ≥90^th^ percentile^a^**  **N=1 032** | **HOMA-IR  <90^th^ percentile^a^**  **N=1 839** | **HOMA-IR  ≥90^th^ percentile^a^**  **N=382** |
| --- | --- | --- | --- | --- |
| Age, mean (SD) | 7.5 (1.9) | 8.2 (1.7) | 7.8 (1.9) | 8.1 (1.8) |
| Girls, N (%) | 1 454 (50.7) | 526 (51.0) | 911 (49.5) | 206 (53.9) |
| Country, N (%) |  |  |  |  |
| Italy | 392 (13.7) | 380 (36.8) | 334 (18.2) | 143 (37.4) |
| Estonia | 656 (22.9) | 149 (14.4) | 432 (23.5) | 78 (20.4) |
| Cyprus | 313 (10.9) | 162 (15.7) | 83 (4.5) | 17 (4.5) |
| Belgium | 88 (3.1) | 9 (0.9) | 79 (4.3) | 4 (1.1) |
| Sweden | 426 (14.9) | 83 (8.0) | 255 (13.9) | 24 (6.3) |
| Germany | 358 (12.5) | 64 (6.2) | 140 (7.6) | 12 (3.1) |
| Hungary | 403 (14.1) | 131 (12.7) | 306 (16.6) | 84 (22.0) |
| Spain | 232 (8.1) | 54 (5.2) | 210 (11.4) | 20 (5.2) |
| Highest level of parental education, N (%)^b^ |  |  |  |  |
| Low | 84 (2.9) | 97 (9.4) | 71 (3.9) | 35 (9.2) |
| Medium | 1 119 (39.0) | 520 (50.4) | 743 (40.4) | 189 (49.5) |
| High | 1 665 (58.1) | 415 (40.2) | 1 025 (55.7) | 158 (41.4) |
| Well-being score, median (IQR) | 40 (37-43) | 39 (36-42) | 40 (37-43) | 39.5 (36-42) |
| Napping (yes), N (%) | 655 (22.8) | 173 (16.8) | 370 (20.1) | 81 (21.2) |
| Average napping time (minutes per day), median (IQR)^c^ | 86 (43-103) | 64 (34-90) | 86 (43-99) | 86 (43-90) |
| Nocturnal sleep duration (hours), mean (SD) |  |  |  |  |
| Weekly average | 9.86 (0.80) | 9.61 (0.71) | 9.82 (0.76) | 9.65 (0.70) |
| Weekday | 9.74 (0.87) | 9.48 (0.77) | 9.69 (0.83) | 9.47 (0.75) |
| Weekend day | 10.18 (0.98) | 9.96 (0.96) | 10.14 (0.95) | 10.08 (0.99) |

*HOMA-IR* homeostasis model assessment for insulin resistance, *IQR* interquartile range, *SD* standard deviation

^a^ based on reference values derived from data of normal weight children participating in the IDEFICS/I.Family studies according to previously described methods [1-3]

^b^ categorisation according to the “International Standard Classification of Education” (levels 0-2=low, 3-5=medium and 6-8=high) [4]

^c^ children not having a nap were not considered in this statistic (N=3 072)

**REFERENCES**

1. Nagy P, Kovács É, Moreno LA, Veidebaum T, Tornaritis M, Kourides Y, et al. Percentile reference values for anthropometric body composition indices in European children from the IDEFICS study. Int J Obes (Lond). 2014;38 Suppl 2:S15-25. Epub 2014/09/16. doi: 10.1038/ijo.2014.131. PubMed PMID: 25219408. Erratum in: Int J Obes (Lond). 2016;40(10):1604-5.

2. Peplies J, Jiménez-Pavón D, Savva SC, Buck C, Günther K, Fraterman A, et al. Percentiles of fasting serum insulin, glucose, HbA1c and HOMA-IR in pre-pubertal normal weight European children from the IDEFICS cohort. Int J Obes (Lond). 2014;38 Suppl 2:S39-47. Epub 2014/11/08. doi: 10.1038/ijo.2014.134. PubMed PMID: 25376219.

3. Intemann T, Pohlabeln H, Herrmann D, Ahrens W, Pigeot I. Estimating age- and height-specific percentile curves for children using GAMLSS in the IDEFICS study. In: Wilhelm AF, Kestler HA, editors. Analysis of large and complex data Studies in classification, data analysis, and knowledge organization. Heidelberg: Springer International Publishing; 2016. p. 385-94.

4. UNESCO Institute for Statistics. International Standard Classification of Education ISCED 2011. Montreal, Canada: United Nations Educational, Scientific and Cultural Organisation 2012.
