# Supplementary material for: Associations between sleep duration and insulin resistance in European children and adolescents considering the mediating role of abdominal obesity
Source: PLoS One. 2020 Jun 30;15(6):e0235049. doi: 10.1371/journal.pone.0235049 (PMC7326225; doi:10.1371/journal.pone.0235049)
Supplement: S3 Table — (DOCX) [file pone.0235049.s003.docx]

**S3 Table:** Pearson correlation coefficients and p-values among baseline SLEEP z-score, baseline WAIST z-score, baseline HOMA z-score, WAIST z-score at follow-up and HOMA z-score at follow-up

|  | **SLEEP z-score_baseline_** | **WAIST  z-score_baseline_** | **HOMA  z-score_baseline_** | **WAIST  z-score_FU_** | **HOMA  z-score_FU_** |
| --- | --- | --- | --- | --- | --- |
| **SLEEP z-score_baseline_** | \| 1.00 \| \| --- \| \|  \| \|  \| |  |  |  |  |
| **WAIST  z-score_baseline_** | \| -0.141 \| \| --- \| \| <0.001 \| \| N=3 900 \| | \| 1.00 \| \| --- \| \|  \| \|  \| |  |  |  |
| **HOMA  z-score_baseline_** | \| -0.129 \| \| --- \| \| <0.001 \| \| N=2 221 \| | \| 0.501 \| \| --- \| \| <0.001 \| \| N=2 221 \| | \| 1.00 \| \| --- \| \|  \| \|  \| |  |  |
| **WAIST  z-score_FU_** | \| -0.137 \| \| --- \| \| <0.001 \| \| N=3 900 \| | \| 0.778 \| \| --- \| \| <0.001 \| \| N=3 900 \| | \| 0.430 \| \| --- \| \| <0.001 \| \| N=2 221 \| | \| 1.00 \| \| --- \| \|  \| \|  \| | \|  \| \| --- \| \|  \| \|  \| |
| **HOMA  z-score_FU_** | \| -0.027 \| \| --- \| \| 0.219 \| \| N=2 150 \| | \| 0.313 \| \| --- \| \| <0.001 \| \| N=2 150 \| | \| 0.325 \| \| --- \| \| <0.001 \| \| N=1 319 \| | \| 0.410 \| \| --- \| \| <0.001 \| \| N=2 150 \| | \| 1.00 \| \| --- \| \|  \| \|  \| |

*SLEEP* nocturnal sleep duration, *HOMA* homeostasis model assessment for insulin resistance, *WAIST* waist circumference, *FU* follow-up
