# Supplementary material for: Associations between sleep duration and insulin resistance in European children and adolescents considering the mediating role of abdominal obesity
Source: PLoS One. 2020 Jun 30;15(6):e0235049. doi: 10.1371/journal.pone.0235049 (PMC7326225; doi:10.1371/journal.pone.0235049)
Supplement: S6 Table — (DOCX) [file pone.0235049.s006.docx]

S6 Table: Sensitivity analysis (weekend nocturnal sleep duration) - Indirect and total effects and corresponding p-values obtained from path analysis of cross-sectional and longitudinal associations of weekend nocturnal sleep duration z-score with waist circumference z-score and homeostasis model assessment for insulin resistance z-score

|  | *Whole group (N=3 900)* | |
| --- | --- | --- |
|  | *Unst. estimate* | *p-value* |
| ***Indirect effects*** |  |  |
| WE SLEEP z-score_baseline_ 🡪 WAIST z-score_baseline_ 🡪 HOMA z-score_baseline_ | -0.035 | <0.001 |
| WE SLEEP z-score_baseline_ 🡪 WAIST z-score_baseline_ 🡪 WAIST z-score_FU_ | -0.080 | <0.001 |
| WE SLEEP z-score_baseline_ 🡪 WE SLEEP z-score_FU_ 🡪 WAIST z-score_FU_ | -0.001 | 0.763 |
| WE SLEEP z-score_baseline_ 🡪 WAIST z-score_FU_ 🡪 HOMA z-score_FU_ | -0.006 | 0.194 |
| WE SLEEP z-score_baseline_ 🡪 WAIST z-score_baseline_ 🡪 HOMA z-score_FU_ | 0.008 | 0.011 |
| WE SLEEP z-score_baseline_ 🡪 HOMA z-score_baseline_ 🡪 HOMA z-score_FU_ | 0.006 | 0.127 |
| WE SLEEP z-score_baseline_ 🡪 WE SLEEP z-score_FU_ 🡪 HOMA z-score_FU_ | 0.005 | 0.305 |
| WE SLEEP z-score_baseline_ 🡪 WAIST z-score_baseline_ 🡪 WAIST z-score_FU_ 🡪 HOMA z-score_FU_ | -0.024 | <0.001 |
| WE SLEEP z-score_baseline_ 🡪 WE SLEEP z-score_FU_ 🡪 WAIST z-score_FU_ 🡪 HOMA z-score_FU_ | 0.000 | 0.763 |
| WE SLEEP z-score_baseline_ 🡪 WAIST z-score_baseline_ 🡪 HOMA z-score_baseline_ 🡪 HOMA z-score_FU_ | -0.007 | 0.001 |
| ***Total effects*** |  |  |
| WE SLEEP z-score_baseline_ 🡪 HOMA z-score_baseline_ | -0.003 | 0.908 |
| WE SLEEP z-score_baseline_ 🡪 WAIST z-score_FU_ | -0.101 | <0.001 |
| WE SLEEP z-score_baseline_ 🡪 HOMA z-score_FU_ | -0.012 | 0.606 |

*Unst* unstandardised; *WE SLEEP* weekend nocturnal sleep duration; *WAIST* waist circumference; *HOMA* homeostasis model assessment for insulin resistance; baseline: 2009/10, follow-up (FU): 2013/14; Path model was adjusted for age, sex, country, highest educational level of parents, well-being score, weekend napping time (all at baseline), pubertal status (at FU) and follow-up time
