# Supplementary material for: Associations between sleep duration and insulin resistance in European children and adolescents considering the mediating role of abdominal obesity
Source: PLoS One. 2020 Jun 30;15(6):e0235049. doi: 10.1371/journal.pone.0235049 (PMC7326225; doi:10.1371/journal.pone.0235049)
Supplement: S7 Table — (DOCX) [file pone.0235049.s007.docx]

S7 Table: Sensitivity analysis (complete case analysis) - Indirect and total effects and corresponding p-values obtained from path analysis of cross-sectional and longitudinal associations of nocturnal sleep duration z-score with waist circumference z-score and homeostasis model assessment for insulin resistance z-score

|  | *Whole group (N=1 319)* | |  | *Pre-school children (N=234)* | |  | *School children (N=1 085)* | |
| --- | --- | --- | --- | --- | --- | --- | --- | --- |
|  | *Unst. estimate* | *p-value* |  | *Unst. estimate* | *p-value* |  | *Unst. estimate* | *p-value* |
| ***Indirect effects*** |  |  |  |  |  |  |  |  |
| SLEEP z-score_baseline_ 🡪 WAIST z-score_baseline_ 🡪 HOMA z-score_baseline_ | -0.060 | <0.001 |  | -0.077 | 0.036 |  | -0.057 | 0.001 |
| SLEEP z-score_baseline_ 🡪 WAIST z-score_baseline_ 🡪 WAIST z-score_FU_ | -0.126 | <0.001 |  | -0.182 | 0.035 |  | -0.119 | 0.001 |
| SLEEP z-score_baseline_ 🡪 SLEEP z-score_FU_ 🡪 WAIST z-score_FU_ | -0.006 | 0.411 |  | 0.017 | 0.634 |  | -0.007 | 0.324 |
| SLEEP z-score_baseline_ 🡪 WAIST z-score_FU_ 🡪 HOMA z-score_FU_ | 0.001 | 0.921 |  | -0.008 | 0.801 |  | 0.003 | 0.695 |
| SLEEP z-score_baseline_ 🡪 WAIST z-score_baseline_ 🡪 HOMA z-score_FU_ | 0.009 | 0.121 |  | 0.005 | 0.782 |  | 0.008 | 0.177 |
| SLEEP z-score_baseline_ 🡪 HOMA z-score_baseline_ 🡪 HOMA z-score_FU_ | -0.003 | 0.639 |  | 0.008 | 0.468 |  | -0.007 | 0.323 |
| SLEEP z-score_baseline_ 🡪 SLEEP z-score_FU_ 🡪 HOMA z-score_FU_ | 0.003 | 0.766 |  | 0.030 | 0.319 |  | 0.000 | 0.979 |
| SLEEP z-score_baseline_ 🡪 WAIST z-score_baseline_ 🡪 WAIST z-score_FU_ 🡪 HOMA z-score_FU_ | -0.033 | <0.001 |  | -0.055 | 0.053 |  | -0.029 | 0.002 |
| SLEEP z-score_baseline_ 🡪 SLEEP z-score_FU_ 🡪 WAIST z-score_FU_ 🡪 HOMA z-score_FU_ | -0.002 | 0.409 |  | 0.005 | 0.642 |  | -0.002 | 0.325 |
| SLEEP z-score_baseline_ 🡪 WAIST z-score_baseline_ 🡪 HOMA z-score_baseline_ 🡪 HOMA z-score_FU_ | -0.011 | 0.002 |  | -0.006 | 0.381 |  | -0.012 | 0.003 |
| ***Total effects*** |  |  |  |  |  |  |  |  |
| SLEEP z-score_baseline_ 🡪 HOMA z-score_baseline_ | -0.074 | 0.026 |  | 0.014 | 0.872 |  | -0.088 | 0.014 |
| SLEEP z-score_baseline_ 🡪 WAIST z-score_FU_ | -0.129 | 0.003 |  | -0.191 | 0.120 |  | -0.113 | 0.016 |
| SLEEP z-score_baseline_ 🡪 HOMA z-score_FU_ | -0.022 | 0.502 |  | -0.085 | 0.389 |  | -0.010 | 0.767 |

*Unst.* unstandardised; *SLEEP* nocturnal sleep duration; *WAIST* waist circumference; *HOMA* homeostasis model assessment for insulin resistance; baseline: 2009/10, follow-up (FU): 2013/14; Path model was adjusted for age, sex, country, highest educational level of parents, well-being score, average napping time (all at baseline), pubertal status (at FU) and follow-up time
