# Supplementary material for: Associations between sleep duration and insulin resistance in European children and adolescents considering the mediating role of abdominal obesity
Source: PLoS One. 2020 Jun 30;15(6):e0235049. doi: 10.1371/journal.pone.0235049 (PMC7326225; doi:10.1371/journal.pone.0235049)
Supplement: S8 Table — (DOCX) [file pone.0235049.s008.docx]

S8 Table: Sensitivity analysis (HOMA at baseline and/or follow-up) - Indirect and total effects and corresponding p-values obtained from path analysis of cross-sectional and longitudinal associations of nocturnal sleep duration z-score with waist circumference z-score and homeostasis model assessment for insulin resistance z-score

|  | *Whole group (N=3 052)* | |  | *Pre-school children (N=594)* | |  | *School children (N=2 458)* | |
| --- | --- | --- | --- | --- | --- | --- | --- | --- |
|  | *Unst. estimate* | *p-value* |  | *Unst. estimate* | *p-value* |  | *Unst. estimate* | *p-value* |
| ***Indirect effects*** |  |  |  |  |  |  |  |  |
| SLEEP z-score_baseline_ 🡪 WAIST z-score_baseline_ 🡪 HOMA z-score_baseline_ | -0.042 | <0.001 |  | -0.030 | 0.107 |  | -0.043 | <0.001 |
| SLEEP z-score_baseline_ 🡪 WAIST z-score_baseline_ 🡪 WAIST z-score_FU_ | -0.096 | <0.001 |  | -0.080 | 0.106 |  | -0.096 | <0.001 |
| SLEEP z-score_baseline_ 🡪 SLEEP z-score_FU_ 🡪 WAIST z-score_FU_ | -0.003 | 0.586 |  | 0.013 | 0.468 |  | -0.003 | 0.478 |
| SLEEP z-score_baseline_ 🡪 WAIST z-score_FU_ 🡪 HOMA z-score_FU_ | -0.002 | 0.789 |  | 0.002 | 0.931 |  | 0.000 | 0.937 |
| SLEEP z-score_baseline_ 🡪 WAIST z-score_baseline_ 🡪 HOMA z-score_FU_ | 0.010 | 0.013 |  | 0.014 | 0.191 |  | 0.007 | 0.085 |
| SLEEP z-score_baseline_ 🡪 HOMA z-score_baseline_ 🡪 HOMA z-score_FU_ | -0.001 | 0.793 |  | 0.004 | 0.538 |  | -0.004 | 0.460 |
| SLEEP z-score_baseline_ 🡪 SLEEP z-score_FU_ 🡪 HOMA z-score_FU_ | 0.007 | 0.309 |  | 0.012 | 0.567 |  | 0.006 | 0.361 |
| SLEEP z-score_baseline_ 🡪 WAIST z-score_baseline_ 🡪 WAIST z-score_FU_ 🡪 HOMA z-score_FU_ | -0.029 | <0.001 |  | -0.035 | 0.116 |  | -0.025 | <0.001 |
| SLEEP z-score_baseline_ 🡪 SLEEP z-score_FU_ 🡪 WAIST z-score_FU_ 🡪 HOMA z-score_FU_ | -0.001 | 0.585 |  | 0.006 | 0.474 |  | -0.001 | 0.478 |
| SLEEP z-score_baseline_ 🡪 WAIST z-score_baseline_ 🡪 HOMA z-score_baseline_ 🡪 HOMA z-score_FU_ | -0.008 | 0.001 |  | -0.002 | 0.450 |  | -0.009 | 0.001 |
| ***Total effects*** |  |  |  |  |  |  |  |  |
| SLEEP z-score_baseline_ 🡪 HOMA z-score_baseline_ | -0.048 | 0.054 |  | 0.019 | 0.745 |  | -0.061 | 0.027 |
| SLEEP z-score_baseline_ 🡪 WAIST z-score_FU_ | -0.104 | <0.001 |  | -0.062 | 0.414 |  | -0.101 | 0.002 |
| SLEEP z-score_baseline_ 🡪 HOMA z-score_FU_ | -0.017 | 0.501 |  | -0.030 | 0.691 |  | -0.009 | 0.727 |

*Unst.* unstandardised; *SLEEP* nocturnal sleep duration; *WAIST* waist circumference; *HOMA* homeostasis model assessment for insulin resistance; baseline: 2009/10, follow-up (FU): 2013/14; Path model was adjusted for age, sex, country, highest educational level of parents, well-being score, average napping time (all at baseline), pubertal status (at FU) and follow-up time
