# Supplementary material for: Associations between sleep duration and insulin resistance in European children and adolescents considering the mediating role of abdominal obesity
Source: PLoS One. 2020 Jun 30;15(6):e0235049. doi: 10.1371/journal.pone.0235049 (PMC7326225; doi:10.1371/journal.pone.0235049)
Supplement: S9 Table — (DOCX) [file pone.0235049.s009.docx]

S9 Table: Additional analysis for investigating potential u-shaped associations - Multilevel regression models investigating the cross-sectional associations between baseline sleep duration categories (sleep duration z-score according to quartiles), baseline waist circumference z-score and baseline homeostasis model assessment for insulin resistance z-score

|  | WAIST z-score_baseline_ (N=3 900) | | |  | HOMA z-score_baseline_ (N=2 221)* | | |  |
| --- | --- | --- | --- | --- | --- | --- | --- | --- |
|  | *β* | *95% CI* | *p-value* |  | *β* | *95% CI* | *p-value* |  |
| SLEEP z-score_baseline_ |  |  |  |  |  |  |  |  |
| 1^st^ quartile (N=959) | 0.206 | 0.077; 0.335 | 0.002 |  | 0.103 | -0.029; 0.235 | 0.117 |  |
| 2^nd^ quartile (N=992) | 0.174 | 0.050; 0.299 | 0.007 |  | 0.037 | -0.090; 0.163 | 0.546 |  |
| 3^rd^ quartile (ref) (N=963) | 0 |  |  |  | 0 |  |  |  |
| 4^th^ quartile (N=986) | -0.059 | -0.191; 0.072 | 0.371 |  | -0.025 | -0.160; 0.110 | 0.702 |  |

*SLEEP* sleep duration*; WAIST* waist circumference; *HOMA* homeostasis model assessment; *CI* confidence interval; Both models were adjusted for age, sex, country, highest educational level of parents, napping time and well-being score (all at baseline) and pubertal status (at follow-up) and included a random effect for family affiliation; *missing: 1 679 (1^st^ quartile SLEEP z-score_baseline_: 428, 2^nd^ quartile: 434, 3^rd^ quartile: 356, 4^th^ quartile: 461)
