# Supplementary material for: Associations between sleep duration and insulin resistance in European children and adolescents considering the mediating role of abdominal obesity
Source: PLoS One. 2020 Jun 30;15(6):e0235049. doi: 10.1371/journal.pone.0235049 (PMC7326225; doi:10.1371/journal.pone.0235049)
Supplement: S10 Table — (DOCX) [file pone.0235049.s010.docx]

S10 Table: Sensitivity analysis (additional adjustment for residence in the intervention vs. control region) - Indirect and total effects and corresponding p-values obtained from path analysis of cross-sectional and longitudinal associations of nocturnal sleep duration z-score with waist circumference z-score and homeostasis model assessment for insulin resistance z-score

|  | *Whole group (N=3 900)** | |
| --- | --- | --- |
|  | *Unst. estimate* | *p-value* |
| ***Indirect effects*** |  |  |
| SLEEP z-score_baseline_ 🡪 WAIST z-score_baseline_ 🡪 HOMA z-score_baseline_ | -0.042 | <0.001 |
| SLEEP z-score_baseline_ 🡪 WAIST z-score_baseline_ 🡪 WAIST z-score_FU_ | -0.094 | <0.001 |
| SLEEP z-score_baseline_ 🡪 SLEEP z-score_FU_ 🡪 WAIST z-score_FU_ | -0.006 | 0.242 |
| SLEEP z-score_baseline_ 🡪 WAIST z-score_FU_ 🡪 HOMA z-score_FU_ | 0.001 | 0.885 |
| SLEEP z-score_baseline_ 🡪 WAIST z-score_baseline_ 🡪 HOMA z-score_FU_ | 0.008 | 0.030 |
| SLEEP z-score_baseline_ 🡪 HOMA z-score_baseline_ 🡪 HOMA z-score_FU_ | -0.001 | 0.769 |
| SLEEP z-score_baseline_ 🡪 SLEEP z-score_FU_ 🡪 HOMA z-score_FU_ | 0.006 | 0.417 |
| SLEEP z-score_baseline_ 🡪 WAIST z-score_baseline_ 🡪 WAIST z-score_FU_ 🡪 HOMA z-score_FU_ | -0.028 | <0.001 |
| SLEEP z-score_baseline_ 🡪 SLEEP z-score_FU_ 🡪 WAIST z-score_FU_ 🡪 HOMA z-score_FU_ | -0.002 | 0.242 |
| SLEEP z-score_baseline_ 🡪 WAIST z-score_baseline_ 🡪 HOMA z-score_baseline_ 🡪 HOMA z-score_FU_ | -0.008 | 0.001 |
| ***Total effects*** |  |  |
| SLEEP z-score_baseline_ 🡪 HOMA z-score_baseline_ | -0.050 | 0.061 |
| SLEEP z-score_baseline_ 🡪 WAIST z-score_FU_ | -0.096 | <0.001 |
| SLEEP z-score_baseline_ 🡪 HOMA z-score_FU_ | -0.023 | 0.403 |

*Unst.* unstandardised; *SLEEP* nocturnal sleep duration; *WAIST* waist circumference; *HOMA* homeostasis model assessment for insulin resistance; baseline: 2009/10, follow-up (FU): 2013/14; Path model was adjusted for age, sex, country, highest educational level of parents, well-being score, average napping time, indicator for residence in the intervention vs. control region (all at baseline), pubertal status (at FU) and follow-up time; *children not participating in 2007/08 (N=570) were excluded from this analysis
