# Supplementary material for: Associations between sleep duration and insulin resistance in European children and adolescents considering the mediating role of abdominal obesity
Source: PLoS One. 2020 Jun 30;15(6):e0235049. doi: 10.1371/journal.pone.0235049 (PMC7326225; doi:10.1371/journal.pone.0235049)
Supplement: S12 Table — (DOCX) [file pone.0235049.s012.docx]

S12 Table: Sensitivity analysis (adjustment for either Tanner stage or menarche/voice mutation) - Indirect and total effects and corresponding p-values obtained from path analysis of cross-sectional and longitudinal associations of nocturnal sleep duration z-score with waist circumference z-score and homeostasis model assessment for insulin resistance z-score

|  | *Adjustment for Tanner stages (N=2 999)* | |  | *Adjustment for menarche/voice mutation  (N=2 999)* | |
| --- | --- | --- | --- | --- | --- |
|  | *Unst. estimate* | *p-value* |  | *Unst. estimate* | *p-value* |
| ***Indirect effects*** |  |  |  |  |  |
| SLEEP z-score_baseline_ 🡪 WAIST z-score_baseline_ 🡪 HOMA z-score_baseline_ | -0.042 | <0.001 |  | -0.043 | <0.001 |
| SLEEP z-score_baseline_ 🡪 WAIST z-score_baseline_ 🡪 WAIST z-score_FU_ | -0.098 | <0.001 |  | -0.099 | <0.001 |
| SLEEP z-score_baseline_ 🡪 SLEEP z-score_FU_ 🡪 WAIST z-score_FU_ | 0.001 | 0.915 |  | 0.000 | 0.924 |
| SLEEP z-score_baseline_ 🡪 WAIST z-score_FU_ 🡪 HOMA z-score_FU_ | -0.001 | 0.806 |  | -0.002 | 0.766 |
| SLEEP z-score_baseline_ 🡪 WAIST z-score_baseline_ 🡪 HOMA z-score_FU_ | 0.008 | 0.053 |  | 0.008 | 0.047 |
| SLEEP z-score_baseline_ 🡪 HOMA z-score_baseline_ 🡪 HOMA z-score_FU_ | 0.000 | 0.941 |  | 0.000 | 0.952 |
| SLEEP z-score_baseline_ 🡪 SLEEP z-score_FU_ 🡪 HOMA z-score_FU_ | 0.006 | 0.443 |  | 0.005 | 0.488 |
| SLEEP z-score_baseline_ 🡪 WAIST z-score_baseline_ 🡪 WAIST z-score_FU_ 🡪 HOMA z-score_FU_ | -0.025 | <0.001 |  | -0.027 | <0.001 |
| SLEEP z-score_baseline_ 🡪 SLEEP z-score_FU_ 🡪 WAIST z-score_FU_ 🡪 HOMA z-score_FU_ | 0.000 | 0.915 |  | 0.000 | 0.924 |
| SLEEP z-score_baseline_ 🡪 WAIST z-score_baseline_ 🡪 HOMA z-score_baseline_ 🡪 HOMA z-score_FU_ | -0.007 | 0.002 |  | -0.007 | 0.002 |
| ***Total effects*** |  |  |  |  |  |
| SLEEP z-score_baseline_ 🡪 HOMA z-score_baseline_ | -0.044 | 0.132 |  | -0.044 | 0.130 |
| SLEEP z-score_baseline_ 🡪 WAIST z-score_FU_ | -0.102 | <0.001 |  | -0.105 | <0.001 |
| SLEEP z-score_baseline_ 🡪 HOMA z-score_FU_ | -0.004 | 0.880 |  | -0.007 | 0.817 |

*Unst.* unstandardised; *SLEEP* nocturnal sleep duration; *WAIST* waist circumference; *HOMA* homeostasis model assessment for insulin resistance; baseline: 2009/10, follow-up (FU): 2013/14; Path model was adjusted for age, sex, country (in the model using data of children living in the intervention region Hungary and Germany were collapsed into one category because of estimation problems), highest educational level of parents, well-being score, average napping time (all at baseline), pubertal status based on Tanner stages or menarche/voice mutation (at FU) and follow-up time
