# Supplementary material for: Associations between sleep duration and insulin resistance in European children and adolescents considering the mediating role of abdominal obesity
Source: PLoS One. 2020 Jun 30;15(6):e0235049. doi: 10.1371/journal.pone.0235049 (PMC7326225; doi:10.1371/journal.pone.0235049)
Supplement: S1 Fig — (DOCX) [file pone.0235049.s013.docx]

HOMA z-score
_FU_

0.200; p<0.001

-0.068; p=0.014

0.284; p<0.001

0.343; p<0.001

0.016; p=0.566

0.790; p<0.001

WAIST z-score
_FU_

WAIST z-score _baseline_

HOMA z-score
_baseline_

0.025; p=0.365

-0.026; p=0.123

-0.006; p=0.815

-0.102; p<0.001

-0.008; p=0.685

SLEEP z-score _FU_

SLEEP z-score _baseline_

0.274; p<0.001

S1 Figure: Sensitivity analysis (additional adjustment for lifestyle factors) – Path model for the associations of nocturnal sleep duration (SLEEP) z-score with waist circumference (WAIST) z-score and homeostasis model assessment for insulin resistance (HOMA) z-score adjusted for age, sex, country, highest educational level of parents, well-being score, average napping time, fruit and vegetable consumption frequencies (times/week), sports club physical activity (hours/week), duration of electronic media use (hours/week) (all at baseline), pubertal status (at follow-up [FU]) and follow-up time: Unstandardised direct effect estimates and p-values (N=3 239); baseline: 2009/10, FU: 2013/14
