# Supplementary material for: Associations between sleep duration and insulin resistance in European children and adolescents considering the mediating role of abdominal obesity
Source: PLoS One. 2020 Jun 30;15(6):e0235049. doi: 10.1371/journal.pone.0235049 (PMC7326225; doi:10.1371/journal.pone.0235049)
Supplement: S2 Fig — (DOCX) [file pone.0235049.s014.docx]

HOMA z-score
_FU_

0.187; p<0.001

-0.080 p=0.002

0.300; p<0.001

0.344; p<0.001

0.007; p=0.794

0.786; p<0.001

WAIST z-score
_FU_

WAIST z-score _baseline_

HOMA z-score
_baseline_

0.019; p=0.441

-0.025; p=0.126

-0.028; p=0.235

-0.100; p<0.001

0.002; p=0.932

WD SLEEP
z-score _FU_

WD SLEEP
z-score _baseline_

0.274; p<0.001

S2 Figure: Sensitivity analysis (weekday nocturnal sleep duration) - Path model for the association of weekday nocturnal sleep duration (WD SLEEP) z-score with waist circumference (WAIST) z-score and homeostasis model assessment for insulin resistance (HOMA) z-score adjusted for age, sex, country, highest educational level of parents, well-being score, weekday napping time (all at baseline), pubertal status (at follow-up [FU]) and follow-up time: Unstandardised direct effect estimates and p-values (N=3 900); baseline: 2009/10, FU: 2013/14
