# Supplementary material for: Associations between sleep duration and insulin resistance in European children and adolescents considering the mediating role of abdominal obesity
Source: PLoS One. 2020 Jun 30;15(6):e0235049. doi: 10.1371/journal.pone.0235049 (PMC7326225; doi:10.1371/journal.pone.0235049)
Supplement: S3 Fig — (DOCX) [file pone.0235049.s015.docx]

HOMA z-score
_FU_

0.187; p<0.001

-0.081; p=0.002

0.300; p<0.001

0.347; p<0.001

0.005; p=0.825

0.785; p<0.001

WAIST z-score
_FU_

WAIST z-score _baseline_

HOMA z-score
_baseline_

0.023; p=0.301

-0.005; p=0.763

0.033; p=0.115

-0.102; p<0.001

-0.021; p=0.190

WE SLEEP
z-score _FU_

WE SLEEP
z-score _baseline_

0.221; p<0.001

S3 Figure: Sensitivity analysis (weekend nocturnal sleep duration) - Path model for the association of weekend nocturnal sleep duration (WE SLEEP) z-score with waist circumference (WAIST) z-score and homeostasis model assessment for insulin resistance (HOMA) z-score adjusted for age, sex, country, highest educational level of parents, well-being score, weekend napping time (all at baseline), pubertal status (at follow-up [FU]) and follow-up time: Unstandardised direct effect estimates and p-values (N=3 900); baseline: 2009/10, FU: 2013/14
