# Supplementary material for: Associations between sleep duration and insulin resistance in European children and adolescents considering the mediating role of abdominal obesity
Source: PLoS One. 2020 Jun 30;15(6):e0235049. doi: 10.1371/journal.pone.0235049 (PMC7326225; doi:10.1371/journal.pone.0235049)
Supplement: S4 Fig — (DOCX) [file pone.0235049.s016.docx]

1) 0.182; p<0.001

2) 0.083; p=0.310

3) 0.212; p<0.001

HOMA z-score
_FU_

1) 0.364; p<0.001

2) 0.331; p<0.001

3) 0.367; p<0.001

1) 0.263; p<0.001

2) 0.304; p<0.001

3) 0.246; p<0.001

1) -0.052; p=0.086

2) -0.020; p=0.783

3) -0.050; p=0.133

1) 0.014; p=0.663

2) -0.063; p=0.517

3) 0.029; p=0.404

1) 0.767; p<0.001

2) 0.785; p<0.001

3) 0.769; p<0.001

WAIST z-score
_FU_

WAIST z-score _baseline_

HOMA z-score
_baseline_

1) -0.014; p=0.638

2) 0.091; p=0.288

3) -0.031; p=0.316

1) 0.009; p=0.765

2) 0.065; p=0.306

3) -0.001; p=0.979

1) -0.164; p<0.001

2) -0.232; p=0.035

3) -0.154; p=0.001

1) -0.022; p=0.408

2) 0.036; p=0.631

3) -0.027; p=0.316

1) 0.003; p=0.921

2) -0.025; p=0.799

3) 0.012; p=0.696

SLEEP z-score _FU_

SLEEP z-score _baseline_

1) 0.291; p<0.001

2) 0.463; p<0.001

3) 0.261; p<0.001

S4 Figure: Sensitivity analysis (complete case analysis) - Path model for the association of nocturnal sleep duration (SLEEP) z-score with waist circumference (WAIST) z-score and homeostasis model assessment for insulin resistance (HOMA) z-score adjusted for age, sex, country, highest educational level of parents, well-being score, average napping time (all at baseline), pubertal status (at follow-up [FU]) and follow-up time: Unstandardised direct effect estimates and p-values; 1) = Whole group (N=1 319), 2) = Pre-school children (N=234), 3) = School children (N=1 085); baseline: 2009/10, FU: 2013/14
