# Supplementary material for: Associations between sleep duration and insulin resistance in European children and adolescents considering the mediating role of abdominal obesity
Source: PLoS One. 2020 Jun 30;15(6):e0235049. doi: 10.1371/journal.pone.0235049 (PMC7326225; doi:10.1371/journal.pone.0235049)
Supplement: S5 Fig — (DOCX) [file pone.0235049.s017.docx]

1) 0.187; p<0.001

2) 0.078; p=0.377

3) 0.218; p<0.001

HOMA z-score
_FU_

1) 0.345; p<0.001

2) 0.299; p<0.001

3) 0.351; p<0.001

1) 0.300; p<0.001

2) 0.430; p<0.001

3) 0.258; p<0.001

1) -0.080; p=0.002

2) -0.138; p=0.024

3) -0.055; p=0.051

1) 0.006; p=0.805

2) -0.030; p=0.651

3) 0.017; p=0.525

1) 0.787; p<0.001

2) 0.793; p<0.001

3) 0.790; p<0.001

WAIST z-score
_FU_

WAIST z-score _baseline_

HOMA z-score
_baseline_

1) -0.006; p=0.792

2) 0.049; p=0.389

3) -0.019; p=0.456

1) 0.025; p=0.305

2) 0.032; p=0.564

3) 0.024; p=0.358

1) -0.122; p<0.001

2) -0.101; p=0.107

3) -0.122; p<0.001

1) -0.009; p=0.585

2) 0.035; p=0.463

3) -0.013; p=0.477

1) -0.005; p=0.789

2) 0.005; p=0.931

3) -0.002; p=0.937

SLEEP z-score _FU_

SLEEP z-score _baseline_

1) 0.278; p<0.001

2) 0.376; p<0.001

3) 0.257; p<0.001

S5 Figure: Sensitivity analysis (HOMA at baseline and/or follow-up [FU]) - Path model for the association of nocturnal sleep duration (SLEEP) z-score with waist circumference (WAIST) z-score and homeostasis model assessment for insulin resistance (HOMA) z-score adjusted for age, sex, country, highest educational level of parents, well-being score, average napping time (all at baseline), pubertal status (at FU) and follow-up time: Unstandardised direct effect estimates and p-values; 1) = Whole group (N=3 052), 2) = Pre-school children (N=594), 3) = School children (N=2 458); baseline: 2009/10, FU: 2013/14
