# Supplementary material for: Associations between sleep duration and insulin resistance in European children and adolescents considering the mediating role of abdominal obesity
Source: PLoS One. 2020 Jun 30;15(6):e0235049. doi: 10.1371/journal.pone.0235049 (PMC7326225; doi:10.1371/journal.pone.0235049)
Supplement: S6 Fig — (DOCX) [file pone.0235049.s018.docx]

HOMA z-score
_FU_

0.194; p<0.001

-0.071; p=0.012

0.300; p<0.001

0.358; p<0.001

0.001; p=0.963

0.789; p<0.001

WAIST z-score
_FU_

WAIST z-score _baseline_

HOMA z-score
_baseline_

0.021; p=0.415

-0.020; p=0.239

-0.007; p=0.768

-0.119; p<0.001

0.003; p=0.885

SLEEP z-score _FU_

SLEEP z-score _baseline_

0.282; p<0.001

S6 Figure: Sensitivity analysis (additional adjustment for residence in the intervention vs. control region) – Path model for the associations of nocturnal sleep duration (SLEEP) z-score with waist circumference (WAIST) z-score and homeostasis model assessment for insulin resistance (HOMA) z-score adjusted for age, sex, country, highest educational level of parents, well-being score, average napping time, indicator for residence in the intervention vs. control region (all at baseline), pubertal status (at follow-up [FU]) and follow-up time: Unstandardised direct effect estimates and p-values (N=3 330)*; baseline: 2009/10, FU: 2013/14

*children not participating in 2007/08 (N=570) were excluded from this analysis
