# Supplementary material for: Associations between sleep duration and insulin resistance in European children and adolescents considering the mediating role of abdominal obesity
Source: PLoS One. 2020 Jun 30;15(6):e0235049. doi: 10.1371/journal.pone.0235049 (PMC7326225; doi:10.1371/journal.pone.0235049)
Supplement: S7 Fig — (DOCX) [file pone.0235049.s019.docx]

1) -0.022; p=0.622

2) 0.031; p=0.326

3) 0.003; p=0.922

S7 Figure: Sensitivity analysis (stratified by residence in the intervention vs. control region) – Path model for the associations of nocturnal sleep duration (SLEEP) z-score with waist circumference (WAIST) z-score and homeostasis model assessment for insulin resistance (HOMA) z-score adjusted for age, sex, country, highest educational level of parents, well-being score, average napping time (all at baseline), pubertal status (at follow-up [FU]) and follow-up time: Unstandardised direct effect estimates and p-values (N=3 330)*; 1) = Children living in the intervention region (N=1 703); 2) = Children living in the control region (N=1 627); 3) = Whole group (N=3 330); baseline: 2009/10, FU: 2013/14

*children not participating in 2007/08 (N=570) were excluded from this analysis

1) 0.064; p=0.116

2) -0.019; p=0.516

3) 0.023; p=0.363

1) -0.017; p=0.481

2) -0.030; p=0.235

3) -0.020; p=0.240

1) -0.011; p=0.722

2) 0.005; p=0.860

3) 0.002; p=0.904

1) -0.103; p=0.008

2) -0.135; p<0.001

3) -0.119; p<0.001

1) -0.023; p=0.491

2) 0.002; p=0.946

3) -0.007; p=0.762

1) -0.002; p=0.955

2) -0.165; p<0.001

3) -0.072; p=0.012

1) 0.291; p<0.001

2) 0.266; p<0.001

3) 0.282; p<0.001

1) 0.147; p=0.009

2) 0.235; p<0.001

3) 0.194; p<0.001

1) 0.266; p<0.001

2) 0.361; p<0.001

3) 0.298; p<0.001

WAIST z-score
_FU_

1) 0.781; p<0.001

2) 0.805; p<0.001

3) 0.789; p<0.001

1) 0.367; p<0.001

2) 0.352; p<0.001

3) 0.358; p<0.001

WAIST z-score _baseline_

HOMA z-score
_baseline_

HOMA z-score
_FU_

SLEEP z-score _FU_

SLEEP z-score _baseline_
