# Supplementary material for: Associations between sleep duration and insulin resistance in European children and adolescents considering the mediating role of abdominal obesity
Source: PLoS One. 2020 Jun 30;15(6):e0235049. doi: 10.1371/journal.pone.0235049 (PMC7326225; doi:10.1371/journal.pone.0235049)
Supplement: S8 Fig — (DOCX) [file pone.0235049.s020.docx]

1) -0.062; p=0.032

2) -0.064; p=0.026

1) -0.005; p=0.806

2) -0.006; p=0.765

1) 0.286; p<0.001

2) 0.286; p<0.001

1) 0.168; p<0.001

2) 0.169; p<0.001

S8 Figure: Sensitivity analysis (adjustment for either Tanner stage or menarche/voice mutation) – Path model for the associations of nocturnal sleep duration (SLEEP) z-score with waist circumference (WAIST) z-score and homeostasis model assessment for insulin resistance (HOMA) z-score adjusted for age, sex, country , highest educational level of parents, well-being score, average napping time (all at baseline), pubertal status based on Tanner stages or menarche/voice mutation (at follow-up [FU]) and follow-up time: Unstandardised direct effect estimates and p-values; 1) = Adjustment for Tanner stages (N=2 999); 2) = Adjustment for menarche/voice mutation (N=2 999); baseline: 2009/10, FU: 2013/14

1) 0.259; p<0.001

2) 0.270; p<0.001

WAIST z-score
_FU_

1) 0.777; p<0.001

2) 0.785; p<0.001

1) 0.334; p<0.001

2) 0.338; p<0.001

1) 0.016; p=0.568

2) 0.016; p=0.573

WAIST z-score _baseline_

HOMA z-score
_baseline_

HOMA z-score
_FU_

1) -0.126; p<0.001

2) -0.126; p<0.001

1) -0.002; p=0.941

2) -0.002; p=0.952

1) 0.002; p=0.915

2) 0.002; p=0.924

1) 0.020; p=0.441

2) 0.018; p=0.487

SLEEP z-score _FU_

SLEEP z-score _baseline_
