# Supplementary material for: Associations between sleep duration and insulin resistance in European children and adolescents considering the mediating role of abdominal obesity
Source: PLoS One. 2020 Jun 30;15(6):e0235049. doi: 10.1371/journal.pone.0235049 (PMC7326225; doi:10.1371/journal.pone.0235049)
Supplement: Attachment — Submitted filename: Response_to_reviewers_R1.docx [file pone.0235049.s021.docx]

**Point-by-point reply to the academic editor’s and reviewer’s comments**

PONE-D-19-34389: Manuscript “Associations between sleep duration and insulin resistance in European children and adolescents considering the mediating role of abdominal obesity”

We thank the **Academic Editor** for his valuable suggestions. Below we respond to his comments suggesting specific changes of the manuscript.

Academic Editor: 1. Please ensure that your manuscript meets PLOS ONE's style requirements, including those for file naming. The PLOS ONE style templates can be found at http://www.journals.plos.org/plosone/s/file?id=wjVg/PLOSOne_formatting_sample_main_body.pdf and http://www.journals.plos.org/plosone/s/file?id=ba62/PLOSOne_formatting_sample_title_authors_affiliations.pdf

Reply: We have revised the manuscript and named the files accordingly. Our manuscript meets all style requirements now. Please note that we mention the “IDEFICS and I.Family Consortia” next to the twelve individual authors on the manuscript title page. However, we have refrained from providing a list of all members of the consortia (e.g. in the Acknowledgements) as suggested on the “title page style template” because the consortia should not be understood as an author group. Mentioning the consortia in this manner is laid down in the consortia’s publication rules (see http://www.ifamilystudy.eu/wp-content/uploads/2017/03/IDEFICS-IFAMILY_PublRules_2015_09_15.pdf). This addition to the author list was part of several previous publications of the IDEFICS and I.Family Consortia in PLOS ONE as well as in many other journals, as for example the publications by Wolters et al. 2017 (doi: 10.1371/journal.pone.0181485), Wolters et al. 2016 (doi: 10.1371/journal.pone.0165981), Bammann et al. 2014 (doi: 10.1371/journal.pone.0086914) or Gianfagna et al. 2013 (doi: 10.1371/journal.pone.0070632). We therefore hope it is acceptable for PLOS ONE to add the term “on behalf of the IDEFICS and I.Family Consortia”.

Academic Editor: 2. In your Methods section, please provide additional information on the analysis performed. In particular, please clarify whether children who participated in the third (2013/14) examination wave (which is called "follow-up" in the manuscript) all received the intervention. If so, please describe the intervention in the Methods section, and explain whether this was taken into consideration in your longitudinal analysis.

Moreover, please ensure you have provided sufficient details about the participant recruitment method and the demographic details of your participants, such as: a) the recruitment date range (month and year), b) a description of any inclusion/exclusion criteria that were applied to participant recruitment, c) a table of relevant demographic details, d) a statement as to whether your sample can be considered representative of a larger population, e) a description of how participants were recruited, and f) descriptions of where participants were recruited and where the research took place.

Reply: Between the first (2007/08) and the second (2009/10) examination wave of the IDEFICS study a setting-based community-oriented intervention was implemented in selected intervention regions. Approximately 44% of children in our sample lived in the intervention regions (42% lived in the control regions and 15% were newly recruited at the second examination wave). In our manuscript we labelled the second examination wave “baseline” because we did not use data from the first examination wave. This means the intervention took place before the data that were used in the present analysis were collected. Previous analyses have shown that the intervention neither had relevant effects on cardio-metabolic markers nor on sleep duration (Mårild et al., 2015, Michels et al., 2015)^[1]^, i.e. it seems unlikely that the association between sleep duration and waist circumference and HOMA-IR, respectively, could be affected by the intervention. Nevertheless, to study any potential effect of the intervention on our results, in additional analyses we first adjusted our model for residence in the intervention vs. control region and second we conducted an analysis stratified by residence in the intervention vs. control region (including only children who already participated in 2007/08). Neither the additional adjustment altered our results nor did the results markedly differ in children residing in the intervention vs. control regions. These results are now presented as Supporting Information (S6 and S7 Figs, S10 and S11 Tables).

We have added further details on the study design to the Methods section to clarify that not all participants received the intervention:

*“Participants were recruited via a setting-based community-oriented approach in kindergartens and primary schools in two regions in each country; one region was defined as the intervention region, where an intervention for the prevention of childhood obesity was implemented, and the other served as the control region with no intervention [26].”*

Furthermore, we describe the potential impact of the intervention on our results in the Discussion section and include all additional analyses as Supporting Information:

*“Approximately 44% of the children in our study sample lived in the intervention regions. The intervention for the prevention of childhood obesity was implemented between 2007/08 and 2009/10. The present analysis comprised only data collected after the intervention was completed and included children from both regions. The intervention did not reveal any relevant effects on sleep duration, waist circumference and HOMA-IR [57, 58]. However, to preclude any potential effect of the intervention on our results, we conducted additional analyses adjusting the path model for residence in the intervention vs. control region as well as stratifying the model by residence in the intervention vs. control region. This did not markedly change our results (S6 and S7 Figs, S10 and S11 Tables).”*

We also provide the following (additional) information in the Methods section (Study population and procedures) as requested:

a)	*“Children were first examined between September 2007 and May 2008 […]. Subsequently, the intervention was implemented and after its completion, […] children were examined again between September 2009 and June 2010. […] For the I.Family study, children […] were examined again between January 2013 and June 2014.”*

b)	*“Generally, all children attending the kindergartens/primary schools in the selected regions who were between 2 and 9.9 years old were eligible to participate in the study.”*

c)	Demographic details of our study population are presented in Table 1.

d)	*“The regions were selected by convenience, i.e. it was not feasible to obtain nationally representative samples [24]. However, the study followed a population-based approach and study regions were similar to the surrounding regions with respect to their socio-demographic profiles [27].”*

e)	*“Participants were recruited via a setting-based community-oriented approach in kindergartens and primary schools […].”*

f)	*“Participants were recruited […] in kindergartens and primary schools in two regions in each country […]. Usually, examinations took place on site at the kindergartens/primary schools. In addition, in some cases an examination site on the premises of the research centre, a public building or a hospital was established where examinations at kindergartens/schools were not feasible [24].”*

Academic Editor: 3. Our internal editors have looked over your manuscript and determined that it is within the scope of our Determinants, Consequences and Management of Obesity Call for Papers. This collection of papers is headed by a team of Guest Editors for PLOS ONE:Rachel Nugent and Pratibha V. Nerurkar. Additional information can be found on our announcement page: https://collections.plos.org/s/obesity-one.

If you would like your manuscript to be considered for this collection, please let us know in your cover letter and we will ensure that your paper is treated as if you were responding to this call. If you would prefer to remove your manuscript from collection consideration, please specify this in the cover letter.

Reply: Thank you very much for your offer to consider our manuscript as a response to the “Determinants, Consequences and Management of Obesity Call for Papers”. We are happy to accept this kind offer as also expressed in the Cover Letter.

Academic Editor: 4. We note that you have indicated that data from this study are available upon request. PLOS only allows data to be available upon request if there are legal or ethical restrictions on sharing data publicly. For information on unacceptable data access restrictions, please see http://journals.plos.org/plosone/s/data-availability#locunacceptable-data-access-restrictions.

In your revised cover letter, please address the following prompts:

a) If there are ethical or legal restrictions on sharing a de-identified data set, please explain them in detail (e.g., data contain potentially identifying or sensitive patient information) and who has imposed them (e.g., an ethics committee). Please also provide contact information for a data access committee, ethics committee, or other institutional body to which data requests may be sent.

b) If there are no restrictions, please upload the minimal anonymized data set necessary to replicate your study findings as either Supporting Information files or to a stable, public repository and provide us with the relevant URLs, DOIs, or accession numbers. Please see http://www.bmj.com/content/340/bmj.c181.long for guidelines on how to deidentify and prepare clinical data for publication. For a list of acceptable repositories, please see http://journals.plos.org/plosone/s/data-availability#loc-recommended-repositories.

We will update your Data Availability statement on your behalf to reflect the information you provide.

Reply: We are happy to provide more details why our data are only available on request. Please find these details in the Cover Letter.

Academic Editor: 5. We note that you have included the phrase “data not shown” in your manuscript. Unfortunately, this does not meet our data sharing requirements. PLOS does not permit references to inaccessible data. We require that authors provide all relevant data within the paper, Supporting Information files, or in an acceptable, public repository. Please add a citation to support this phrase or upload the data that corresponds with these findings to a stable repository (such as Figshare or Dryad) and provide and URLs, DOIs, or accession numbers that may be used to access these data. Or, if the data are not a core part of the research being presented in your study, we ask that you remove the phrase that refers to these data.

Reply: In the Discussion section we refer to an additional analysis that was conducted to investigate whether there is a potential u-shaped association between i) sleep duration and waist circumference and/or ii) sleep duration and HOMA-IR. Furthermore, we describe in the Discussion section that additional adjustment for residence in the intervention vs. control region did not alter our results. We include the results of these analyses now as Supporting Information and deleted the phrase “data not shown” in both cases:

*“Besides, we conducted additional analyses and did not find evidence for u-shaped associations between sleep duration and both waist circumference and HOMA-IR (S9 Table). […] However, to preclude any potential effect of the intervention on our results, we conducted additional analyses adjusting the path model for residence in the intervention vs. control region as well as stratifying the model by residence in the intervention vs. control region. This did not markedly change our results (S6 and S7 Figs, S10 and S11 Tables).”*

We thank **Reviewer #1** for his/her positive appraisal of our manuscript.

We also thank **Reviewer #2** for his/her valuable comments and suggestions. Below we respond to the major revision points raised.

Reviewer #2: 1) Analyses are based on a subjective measure of sleep duration (patient/parent report on questionnaire). This is a major limitation of the study that can affect reproducibility and the quality of the data. While given the large sample size, it would not be feasible to have performed more objective measures of sleep duration, the authors should elaborate more in the discussion section on how their measure of sleep duration may differ from the gold standard, and how this may impact results and their interpretation.

Reply: We agree that more objective measures of sleep duration would have been preferable. However, as this is not feasible in such large scale studies, we now elaborate in more detail on this limitation:

*“Another limitation of our study is that sleep duration was measured subjectively by questionnaires. Actigraphy has been described as the gold standard for sleep research under natural conditions but was not feasible in our large study for logistical and cost reasons [59]. It has been shown that sleep duration is usually overestimated when obtained by questionnaires in comparison to Actigraphs [60, 61]. Resulting non-differential measurement error of sleep duration may have led to an underestimation of effect sizes.”*

Reviewer #2: 2) They use surrogate markers for insulin resistance (HOMA) and visceral fat (waist circumference). These markers although widely used are not very accurate and the authors need to discuss that.

Reply: We agree that waist circumference and HOMA-IR are only surrogate markers for abdominal obesity and insulin resistance, respectively, and discuss this limitation and consequences:

*“We used waist circumference as a surrogate marker for abdominal obesity that has been found to be highly correlated with visceral adipose tissue measured by magnetic resonance imaging, which is considered a gold standard measure [14]. Further, we used HOMA-IR as a surrogate marker for insulin resistance that has been validated against the hyperinsulinemic-euglycemic clamp, the gold standard measure for determining insulin resistance, and the frequently sampled intravenous glucose tolerance test, which is also considered a valid measure, with good results [62, 63]. The use of waist circumference and HOMA-IR in large-scale epidemiological studies is common and accepted because the superior gold standard measures are time consuming and burdensome for participants and hence not feasible in large population-based studies like ours, particularly not in children [4]. This choice of assessment instruments may introduce random measurement error that may attenuate the strength of any association, but this would hardly reverse the direction of any association observed.”*

Reviewer #2: 3) The definition of puberty is not appropriate because 1) is self-reported and 2) for girls is based on menarche and for boys defined as change in voice. These phenomenon occur 2-3 years after puberty has started. Therefore, the investigators cannot really use puberty as a variable in the study neither they can draw any conclusions on the effect of puberty on the studied phenomenon.

Reply: We agree that self-reported menarche and voice mutation is a rather crude measure of puberty. We had information on breast (girls) and pubic hair (boys) development based on Tanner stages in a subsample of children (N=2 999). Because physical examinations for the assessment of maturation are costly, time-consuming and might lead to a higher number of drop-outs of participants, assessment of puberty by Tanner stages is common practice in large-scale epidemiological studies. In models adjusting for either Tanner stage or menarche/voice mutation, results were very similar and conclusions remained unaltered. We elaborate on this issue in the Discussion section and present the results of the analysis on the subsample as Supporting Information:

*“Pubertal status was self-reported based on menarche and voice mutation. Information on breast (girls) and pubic hair (boys) development based on Tanner stages, which may be considered more accurate, was only available for a subsample (N=2 999). However, adjustment for Tanner stage instead of menarche/voice mutation in this subsample did not alter results (S8 Fig and S12 Table).”*

Reviewer #2: 4) The z-scores were based on their group of patients, rather than on larger healthy population.

Reply: The reference values for waist circumference and HOMA-IR were derived using data from normal weight children participating in the IDEFICS/I.Family study according to the methods described in Nagy et el., Peplies et al. and Intemann et al.^[2]^, i.e. z-scores were not calculated based on the sample in the present study but on the larger IDEFICS/I.Family population of healthy children and adolescents (excluding underweight, overweight and obese participants). Especially with respect to HOMA-IR, we are not aware of any other study to date that was able to provide age- and sex-specific distributions of HOMA-IR in European children and adolescents that might serve as reference values. To our knowledge, only a few studies were conducted in samples from single countries and their use depends on the laboratory assessment method used to determine insulin. In contrast, the IDEFICS/I.Family reference values for HOMA-IR (and also waist circumference) were obtained from a large sample of children/adolescents from eight European countries using a harmonised assessment method and a rigid quality control. Our reference values have been recommended by The Lancet Child & Adolescent Health (https://doi.org/10.1016/S2352-4642(17)30043-3). Therefore, we consider the IDEFICS/I.Family reference values to be the most appropriate for the present study. In the Discussion section we highlight now that the z-scores were derived from normal weight children and adolescents only.

*“In addition, as waist circumference and HOMA-IR are strongly dependent on age and sex, we standardised these parameters using recently developed reference values provided by the IDEFICS/I.Family cohort study based on a huge sample of normal weight children and adolescents from all over Europe.”*

1. Mårild S, Russo P, Veidebaum T, Tornaritis M, De Henauw S, De Bourdeaudhuij I, et al. Impact of a community based health-promotion programme in 2- to 9-year-old children in Europe on markers of the metabolic syndrome, the IDEFICS study. Obes Rev. 2015;16 Suppl 2:41-56.

   Michels N, De Henauw S, Eiben G, Hadjigeorgiou C, Hense S, Hunsberger M, et al. Effect of the IDEFICS multilevel obesity prevention on children's sleep duration. Obes Rev. 2015;16 Suppl 2:68-77.
2. Nagy P, Kovács É, Moreno LA, Veidebaum T, Tornaritis M, Kourides Y, et al. Percentile reference values for anthropometric body composition indices in European children from the IDEFICS study. Int J Obes (Lond). 2014;38 Suppl 2:S15-25.

   Peplies J, Jiménez-Pavón D, Savva SC, Buck C, Günther K, Fraterman A, et al. Percentiles of fasting serum insulin, glucose, HbA1c and HOMA-IR in pre-pubertal normal weight European children from the IDEFICS cohort. Int J Obes (Lond). 2014;38 Suppl 2:S39-47.

   Intemann T, Pohlabeln H, Herrmann D, Ahrens W, Pigeot I. Estimating age- and height-specific percentile curves for children using GAMLSS in the IDEFICS study. In: Wilhelm AF, Kestler HA, editors. Analysis of large and complex data Studies in classification, data analysis, and knowledge organization. Heidelberg: Springer International Publishing; 2016. p. 385-94.
