# Supplementary material for: Associations between sleep duration and insulin resistance in European children and adolescents considering the mediating role of abdominal obesity
Source: PLoS One. 2020 Jun 30;15(6):e0235049. doi: 10.1371/journal.pone.0235049 (PMC7326225; doi:10.1371/journal.pone.0235049)
Supplement: Attachment — Submitted filename: renamed_5094f.docx [file pone.0235049.s022.docx]

**COMMENTS – Manuscript PONE-D-19-34389R1**

Title: “Associations between sleep duration and insulin resistance in European children and adolescents considering the mediating role of abdominal obesity”

**Important note: This review pertains only to ‘statistical aspects’ of the study and so ‘****clinical aspects’ [like medical importance, relevance of the study,** **‘clinical significance and implication(s)’ of the whole study, etc.] are to be evaluated [should be assessed] separately/independently.**

Happy to note that HOMA-IR was standardised according to age and sex (HOMA z-score) [as a Biostatistician I would like you add in brief ‘how done and what effect’]. Since separate reference curves were estimated for HOMA-IR depending on the assessment method used, little more on this would have been appreciated, I guess. How about adding few diagrams (showing ‘path’ including mediating role of abdominal obesity)? [I see that there is lot of material in supplementary files, can some be in main?].

Otherwise the study is absolutely faultless and manuscript/draft of this article communicates the efforts well.

May please be published (with minor revision).
