# Supplementary material for: Associations between sleep duration and insulin resistance in European children and adolescents considering the mediating role of abdominal obesity
Source: PLoS One. 2020 Jun 30;15(6):e0235049. doi: 10.1371/journal.pone.0235049 (PMC7326225; doi:10.1371/journal.pone.0235049)
Supplement: Attachment — Submitted filename: Response_to_reviewers_R2.docx [file pone.0235049.s023.docx]

**Point-by-point reply to reviewer’s comments**

PONE-D-19-34389R1: Manuscript “Associations between sleep duration and insulin resistance in European children and adolescents considering the mediating role of abdominal obesity”

We thank **Reviewer #3** for his valuable comments and suggestions. Below we respond to the revision points raised.

Reviewer #3: Happy to note that HOMA-IR was standardised according to age and sex (HOMA z-score) [as a Biostatistician I would like you add in brief ‘how done and what effect’]. Since separate reference curves were estimated for HOMA-IR depending on the assessment method used, little more on this would have been appreciated, I guess.

Reply: Thank you for this suggestion. We agree that it would be useful to provide further details on the standardisation. As waist circumference was also standardised according to age and sex and is mentioned in the manuscript first, we add further information on the statistical procedure for obtaining the reference values, i.e. the General Additive Model for Location Scale and Shape (GAMLSS) method, to this section:

*“To account for differences in waist circumference by age and sex, we calculated age- and sex-specific z-scores (WAIST z-score). For this purpose, we used percentile curves of waist circumference derived from both the IDEFICS and I.Family populations that were calculated as a function of age stratified by sex using the General Additive Model for Location Scale and Shape (GAMLSS) method [28]. Further details on the application of this method to our data can be found elsewhere [29, 30].”*

Two separate reference curves for HOMA-IR were estimated because the assessment methods for insulin and glucose changed over time in the IDEFICS/I.Family cohort study. We found that especially the assessment method for glucose used in I.Family showed a lower variation in glucose concentrations than the assessment method used in IDEFICS. Hence, the estimation of one HOMA-IR reference curve based on glucose values obtained with the two different methods would have led to an underestimation of the percentages of extreme HOMA z-scores (e.g. those exceeding the 90th percentile) at follow-up. We revised the respective paragraph and explain the rationale for estimating two reference curves as follows:

*“HOMA-IR was standardised according to age and sex (HOMA z-score) using reference values derived from both the IDEFICS and I.Family populations according to previously described methods [30, 32]. As a different assessment method for insulin and glucose was used in the third examination wave, two separate reference curves were estimated for HOMA-IR depending on the assessment method used to account for the lower variation of insulin/glucose measurements in the third examination wave.”*

Reviewer #3: How about adding few diagrams (showing ‘path’ including mediating role of abdominal obesity)? [I see that there is lot of material in supplementary files, can some be in main?]

Reply: In the main manuscript we provide a conceptual framework displaying the hypothesized directions of the associations between sleep duration, waist circumference and HOMA-IR (Figure 2). Furthermore, the direct effects obtained from our main analyses are presented in Figure 3 that also displays the investigated paths between the variables of interest. Supplementary files contain results of sensitivity analyses and the respective figures are the same as Figure 3, except from differences in variables (e.g. weekday sleep duration instead of average sleep duration as the independent variable) or differences in sample size (e.g. complete case analysis). We insert Figure 2 and Figure 3 below and believe that these provide the requested information. However, if this is not the case, please let us know how we can improve the visualisation of the mediating role of abdominal obesity.

Figure 2: Conceptual framework display-ing associations assumed between sleep duration, abdominal obesity and insulin resistance at baseline and follow-up

Figure 3: Path model for the association of nocturnal sleep duration (SLEEP) z-score with waist circumference (WAIST) z-score and homeostasis model assessment (HOMA) for insulin resistance z-score […]
